# A molecular dynamics study on the mechanical response of thermal-pressure rejuvenated Cu_x_Zr_100−x_ metallic glasses

**DOI:** 10.1038/s41598-023-43432-z

**Published:** 2023-09-26

**Authors:** S. Sayad, M. Khanzadeh, Gh. Alahyarizadeh, N. Amigo

**Affiliations:** 1 Faculty of Engineering, Shahid Beheshti University, Tehran, Iran; 2 Departamento de Física, Facultad de Ciencias Naturales, Matemática y del Medio Ambiente, Universidad Tecnológica Metropolitana, Las Palmeras 3360, Ñuñoa 780-0003 Santiago, Chile

**Keywords:** Condensed-matter physics, Structural materials, Theory and computation

## Abstract

A molecular dynamics study was performed on the mechanical response of thermal-pressure rejuvenated Cu_x_Zr_100−x_ metallic glasses. The effect of temperature (50, 300, 600 K) and pressure (0–50 GPa) on the rejuvenation process and the mechanical properties of Cu_x_Zr_100−x_ including stress–strain response, shear localization formation and elastic modulus were investigated. The thermal-pressure rejuvenation process involves transitioning the system to a higher potential energy state and a lower atomic volume, demonstrating the significant influence of pressure on rejuvenation. Our findings reveal that increasing pressure at specific temperatures and material compositions results in reduced yield stress and stress drop. They also indicate that with increasing pressure, the system undergoes a transition towards homogeneity, resulting in enhanced ductility compared to its initial amorphous state. Additionally, high temperatures contribute to lower values of Young's, shear, and bulk moduli, as well as decreased yield stress and stress drop. Consequently, the system becomes more homogeneous, promoting rejuvenation. Furthermore, we observed that the final yield strength of the system increases with higher Cu content for all structures at specific pressures and temperatures. The level of rejuvenation is additionally impacted by the amount of Cu, and structures containing varying content of Cu demonstrate varying degrees of rejuvenation. To validate our findings, we utilized Voronoi analysis, which revealed a higher fraction of densely-packed clusters in the samples. Finally, a total of 10 materials properties were calculated and explored using statistical analysis which shows there are different correlations between pressure, temperature and atomic composition with mechanical properties.

## Introduction

Metallic glasses (MGs) are new alloys with an amorphous structure with many remarkable properties such as high strength and hardness, high elastic strain limit, excellent wear resistance, high corrosion resistance, and good casting ability. Therefore, they are recommended for use in many fields^1–4^. For instance, they are used as medical implants because their resistance to corrosion and oxidation reduces adverse responses in tissues and cells. Other examples involve sports equipment such as skates, skis, knives, and golf clubs due to their wear resistance and high strength^5^. Metallic glasses exhibit improved resistance to corrosion and wear due to their lack of crystalline order^6^. They also possess greater strength in comparison to crystalline materials because of their defect-free nature (i.e. no dislocations)^7^. Nevertheless, the absence of tensile ductility poses a limitation to amorphous metallic alloys, resulting in the catastrophic failure of most MGs under uniaxial tension. Several methods, such as nanoglasses^8,9^, inclusion of crystalline phases^10,11^, and, more recently, thermal and/or mechanical treatments to rejuvenate the samples, have been suggested to counteract this limitation^5^.

Binary MGs are attractive alloys to study from a theoretical point of view due to the simplicity to correlate their mechanical properties to the underlying atomic structure. A remarkable example is the case of CuZr MGs, which have been the subject of extensive research, since CuZr-based MGs possess unique properties and potential applications as those mentioned above^12–14^. Scientists and engineers are interested in understanding the fundamental mechanisms that govern their behavior and properties, as well as exploring ways to further improve their performance. CuZr MGs also have the potential to be used in cutting-edge fields including nanotechnology and healthcare. Because of their biocompatibility and advantageous mechanical properties, Zr-based MGs may, for example, be employed as implant materials^15^. This has led to the development of new theoretical models and experimental techniques that allow for the study and characterization of CuZr MGs at the atomic and nano scales^16–18^. Although many studies have been done on CuZr MGs, it still has research value.

Peng et al. investigated CuZr MGs two-phase deformation and some of its mechanical properties using molecular dynamics (MD) simulation. The results showed that when the yield stress of two-phase MGs is lower than the critical shear stresses required to form a mature shear band, the MGs lead to homogeneous deformation^19^. Chauhan investigated the effect of size and temperature on the mechanical properties of the Cu_50_Zr_50_ structure using MD simulation. It was found that changing the size will not affect the properties of MGs^6^. Yue et al. studied and researched the formation of different structures at different temperatures and strain rates using MD simulation. It was found that the properties of this type of material are strongly affected by strain rate and temperature^4^. Hao investigated the change in the atomic structure of Cu_48_Zr_48_Al_4_ under different temperatures and strain rates. The results show that the yield stress decreases with increasing temperature^20^. Studies at the atomic level indicate that thermal-pressure treatments alter the atomic structure of MGs. In his work, Wang et al. simulated the structural evolution and mechanical response of CuZr with different initial structures under cyclic loading by the MD simulation method. It was found that cyclic loading with different cyclic strain amplitudes can lead to aging or rejuvenating CuZr MG. Additionally, MGs that undergo rapid cooling rates during the quenching process exhibit elevated glass transition temperatures, increased structural heterogeneity, and a greater degree of aging under cyclic loading^6^. Wang et al. rejuvenated Cu-Zr MGs by thermal-pressure method. The present study demonstrated that both cooling rate and pressure exert identical influences on the changes of potential energy ($\Delta PE$) while exerting opposite effects on the changes in atomic volume ($\Delta V$)^21^. Li et al. came to the conclusion that rejuvenation can be carried out at negative pressures and faster cooling rates than the initial quenching process in their investigation on Cu_50_Zr_50_ MG. In other words, MGs may be brought to a higher energy level without changing the nature of the glassy structure^22^. Miyazaki et al.^23^ showed that the application of hydrostatic pressure during quenching considerably boosted both the short-range order (SRO) and medium-range order (MRO), as well as the sample's potential energy (PE), hence improving the samples' flexibility. Feng et al. subsequently published a similar work in which they observed that rejuvenation pressures enhanced plastic behavior despite an increase in the population of full icosahedra (FI), which corresponds to Voronoi polyhedral (VP) with indices $\langle 0,0,12,0\rangle$. This counterintuitive outcome was addressed by demonstrating that FI connection substantially diminished at the MRO as the pressure of rejuvenation rose^24^. Recently, Wang et al. showed that rejuvenation pressures cause CuZr MGs to assume more compact states, therefore lowering the samples' atomic volume. However, they indicated that rejuvenation was accomplished via the production of higher energy levels during thermal-pressure therapies^21,25^. Saida et al.^26^ investigated the thermal rejuvenation in metallic glasses and an increase in the potential energy, a change in the local structure, and mechanical softening were observed after thermal rejuvenation. Pan et al. also investigated the extreme rejuvenation and softening in metallic glasses. They observed a transition to homogeneous after rejuvenation^27^. Regarding the mechanical properties, Zhang et al.^28^ reported decreasing values of shear modulus with temperature, while Brognara et al.^29^ observed increasing values of both the shear and bulk moduli as the copper content increases.

The application of thermal-pressure treatment is a crucial factor in the fabrication process of MGs, as it allows for the formation of these materials. Pressure, in particular, represents an effective and controllable method to modify both the microstructure and energy state of MGs^5^. Constituent atoms can be rearranged into short- or medium-range ordered states^30^. It is commonly accepted that the lack of long-range atomic order in bulk MGs is directly responsible for their unique properties^31^. It is worth noting that MGs with different chemical compositions differ in the ability to form glass and, as a result, the mentioned properties, which are related to the change in density during crystallization and volume expansion^6^. Metallic glasses’ rejuvenation involves structural excitation, which drives the alloys to higher energy states.

Experiments, theoretical, and computer simulations are three standard methods for studying MGs^4^. Here, computer simulation became quite popular and reliable due to the increase in their power^24,32–34^. Molecular dynamics is a computational approach that employs the equation of motion to calculate the temporal positions of atoms as they interact with each other. In this work, according to previous studies, we have investigated the mechanical properties of three different CuZr binary alloys rejuvenated by means of thermal-pressure treatments using MD simulations. To this aim, rejuvenation pressures in the range of 0–50 GPa were considered. In addition, mechanical tests were conducted at temperatures in the range of 50–600 K. This way, statistical correlation can be performed to explore the relationship between atomic composition, mechanical properties, rejuvenation pressure, and temperature. Here, we will first examine how to make MG and simulate MD, and then in the next step, we will analyze and discuss the obtained results. The results are divided into several parts, which are, respectively, compression tests, mechanical properties, changes in PE and atomic volume, and structural changes. Finally, the general conclusion of this work is given.

## Methods

**Figure 1:**
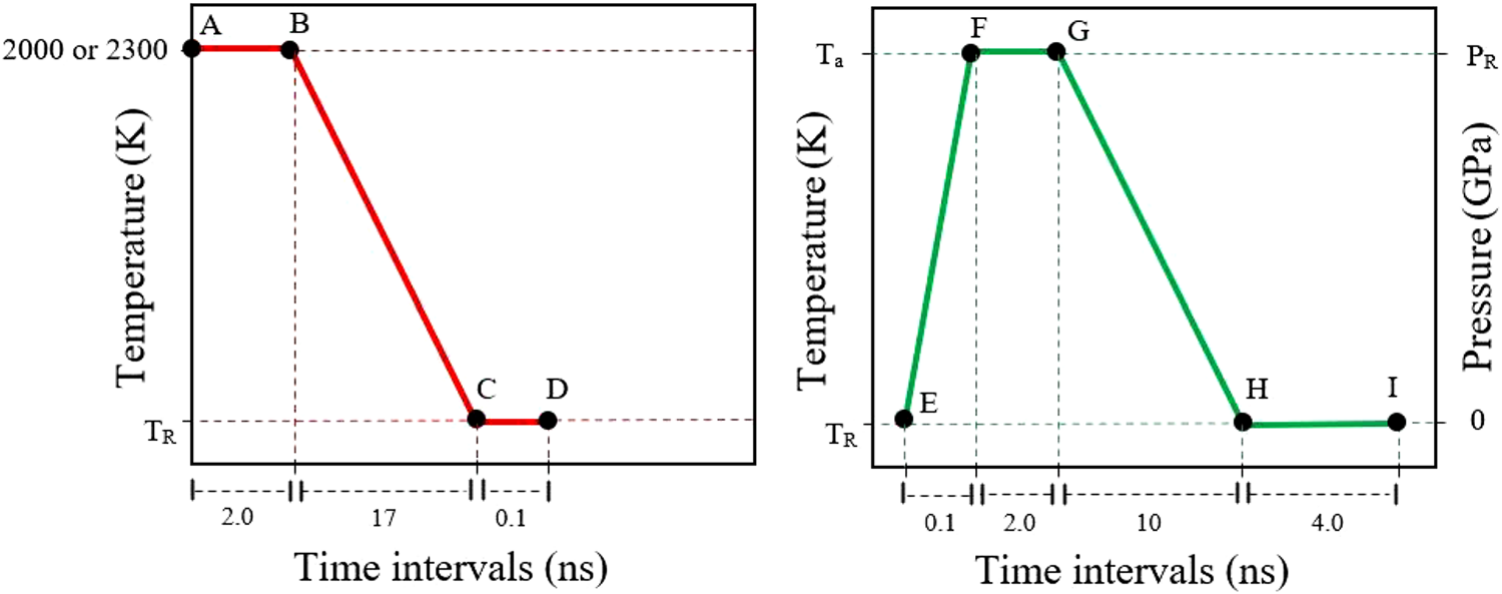
Rejuvenation scheme for the MG samples. The left picture (red) is the process of making MG and the right picture (green) is the process of rejuvenation. P_R_ represents the rejuvenation pressure, which was set at a constant value within the range of 0–50 GPa. T_R_ represents the final desired temperature (50, 300, 600 K) and Ta represents the ~ 1.1–1.3 Tg.

Molecular dynamics simulations were performed using LAMMPS (Large-scale Atomic/Molecular Massively Parallel Simulator) software package^35^. The interatomic potential of Mendelev et al.^36^ was used to investigate the interatomic interactions. The time step was set to 1 fs, and periodic boundary conditions (PBCs) were applied in the *x*, *y*, and *z* directions^37^. The simulations were performed in NPT (constant number, constant pressure, and constant temperature) ensemble^38^. In this work, samples were made with a cooling rate of ${10}^{11}\,\mathrm{K}/\mathrm{s}$ for further investigations. This cooling rate leads to amorphous structures whose radial distribution function is overall consistent with experiments, which is expected since the interatomic potential was checked against x-ray diffraction patterns^36^. The samples were made in the compositions of Cu_64_Zr_36_, Cu_50_Zr_50_, and Cu_46_Zr_54_. The original model contained 6750 atoms. In the initial stage, the samples were subjected to a relaxation process at a temperature of 2000 K and zero pressure for a duration of 2 ns, then the temperature was decreased to three different values, 50, 300 and 600 K (${T}_{R}$), and the model was brought to equilibrium for 0.5 ns under the NPT ensemble to obtain the amorphous system. The temperature for Cu_50_Zr_50_ was 2300 K, because the desired structure was not formed at 2000 K. The preparation scheme for the rejuvenated models is summarized in Fig. 1 in a similar manner as proposed by Amigo et al.^5^.

The rapidly cooled structure was subjected to a heating process until it reached the desired annealing temperature ${T}_{a}$ with a value of $\sim$1.1–1.3 ${T}_{g}$ (where, ${T}_{g}$ is the glass-transition temperature) for 0.1 ns while scaling the pressure up to the target rejuvenation pressure, ${P}_{R}$, (E–F). The value of 1.3 ${T}_{g}$ was chosen since prior research has shown that rejuvenation can be accomplished at annealing temperatures between 1.1 and 1.3 ${T}_{g}$^22–24^. After this, relaxation was carried out at ${T}_{a}$ and ${P}_{R}$ for 2 ns (F–G), and quenching was applied from ${T}_{a}$ to ${T}_{R}$ at ${10}^{11}\,\mathrm{K}/\mathrm{s}$ while decreasing the pressure to 0 GPa (G–H). After a relaxing step at ${T}_{R}$ under zero pressure, the final sample was produced (H–I). All steps were under the NPT ensemble. Four rejuvenated MGs were produced using a variety of rejuvenation pressures, which ranged from 0 to 50 GPa as used previously^5,21,24^. It is worth mentioning that the 0 GPa case can be regarded as non-rejuvenation treatment. Previous studies reported that no rejuvenation can be achieved if the same cooling rate is employed for both obtaining the amorphous sample and quenching from ${T}_{a}$ to ${T}_{R}$^23^. Then the samples were replicated 5 times in the *y* direction for the compression test. For a duration of 0.1 ns, relaxation was carried out at ${T}_{R}$ with PBCs along the *z* and *y* directions, while implementing free boundary conditions (FBCs) along the *x* direction. This relaxation process ensured that the artificial interfaces upon replication were fully relaxed. Notably, the initial temperature for ${T}_{R}=50\,\mathrm{K}$ was set at 300 K and subsequently reduced to 50 K (under the NPT ensemble). While linking the *z*-direction to a zero pressure barostat, all samples were put through compression tests in the *y*-direction. During the uniaxial deformation, the *x*-boundary condition was maintained free. The temperature was kept at ${T}_{R}$ while a ${10}^{8}\,{\mathrm{s}}^{-1}$ compressive strain rate was applied.

The Open Visualization Tool (OVITO) package's local atomic von Mises strain, ${\eta }^{Mises}$^39^, was used to examine the atomic-level deformation of the samples^40^. Following Cheng et al.'s definition^41^, the degree of strain localization, $\Psi$, was measured,

1 $$\Psi = \sqrt {\frac{1}{N}\sum\limits_{i = 1}^{N} {\left( {\eta_{i}^{Mises} - \eta_{ave}^{Mises} } \right)^{2} } } ,$$

where *N* is the total number of atoms, ${\eta }_{i}^{Mises}$ is the von Mises strain of atom *i*, and ${\eta }_{ave}^{Mises}$ is the average local atomic von Mises strain of all atoms.

The Voronoi polyhedra analysis, as implemented in OVITO, was used to characterize the atomic structure. A Voronoi polyhedron's volume was used to compute the per-atom volume^42^.

Elastic constants were calculated by means of small perturbations of the rejuvenated MG. To this aim, the simulation box was slightly deformed under the NVE ensemble using PBCs. Deformation was small enough to ensure an elastic regime. In order to calculate the elastic constants, the components of the virial stress tensor as implemented in LAMMPS were employed. For each rejuvenation pressure value, the elastic tensor ${C}_{ij}$ was identified in this manner. The following set of equations were implemented to determine the terms of ${C}_{ij}$ for the bulk modulus, *B*, and shear modulus, *G*,

2 $$B = \frac{1}{3}(C_{11} + 2C_{12} ),$$

3 $$G = \frac{1}{2}(C_{11} - C_{12} ).$$

The Poisson ratio was obtained using the relationship

4 $$\nu = \frac{{\left( {3 - 2\frac{G}{B}} \right)}}{{\left( {6 + 2\frac{G}{B}} \right)}}$$

## Results

The results of the MD simulations of the rejuvenation process of MGs have been done by examining different results including PE, atomic volume variation and Voronoi analysis. After that, the mechanical response of MGs has been done by examining three different results including strain–stress diagram, degree of strain localization and transition to homogenization (microstructural evolution). In these results, the effects of temperature, pressure and elemental composition were investigated. Also, calculations of elastic coefficients and related mechanical parameters have been used to confirm the mechanical response results.

### Rejuvenation process

One of the factors that affects the mechanical response of MGs is the rejuvenation process. The rejuvenation process involves transitioning the system to a higher PE state. Numerous factors contribute to this process, and in this study, we explore the influence of pressure, temperature, and elemental composition. To assess the degree of rejuvenation, it is crucial to analyze changes in PE, which are closely tied to variations in atomic volume. Therefore, we examine the PE and atomic volume for each sample. We also employ Voronoi analysis to validate the observed changes. The following steps outline the sequential approach to be followed.

#### Potential energy and atomic volume variation

Thermal-pressure treatments have been reported to drive MGs to higher energy states^21^. The average PE per atom, $\overline{U}$, was determined in order to quantify this impact. Since this parameter by itself does not provide valuable information, the change of PE was obtained as the difference of energy

5 $$\Delta \overline{U} = \overline{U} - \overline{{U_{0} }}$$

where $\overline{U}$ and $\overline{{U }_{0}}$ are the average per-atom PE of a sample for a given value of ${P}_{R}$ and for ${P}_{R}=0$, respectively.

**Figure 2:**
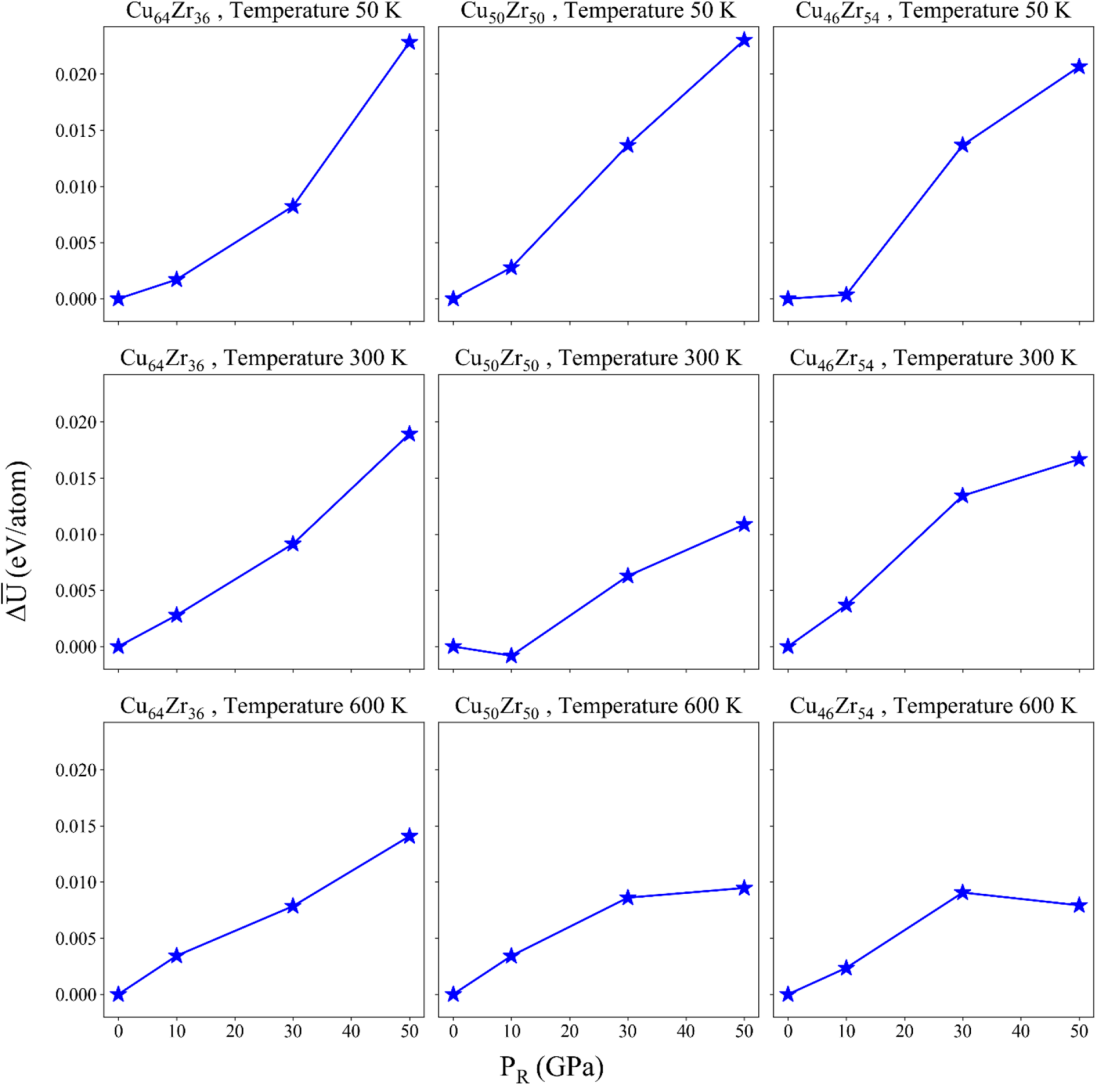
Variation of the average PE per atom for different P_R_ values.

In general, the change in PE of the material indicates the stored energy in its atomic configuration^43^. Variations of PE for different ${P}_{R}$ values applied during the thermal-pressure treatments, are shown in Fig. 2.

As observed, the increase in pressure leads to an increase in $\overline{U}$ of the samples. This increase in PE signifies the intensification of nanoscale defects in the system and a greater disorder in the atomic arrangement of MGs^43^. Specifically, the positive changes in PE resulting from increased pressure indicate that the thermal-pressure method induces structural disordering, or rejuvenation, in the CuZr alloy^43^. This process drives the samples towards a metastable state with higher energy, as previously reported by Amigo et al.^5^, thereby promoting the nucleation of homogeneous shear transformation zones (STZs) when the samples are subjected to external loads. From a PE landscape perspective, the thermal-pressure loading facilitates the transition of MGs from a low-energy state to a high-energy rejuvenated state. In this high-energy rejuvenated state, the lower activation energy for local plastic deformation leads to a greater propensity for the induction of STZs. In the following, it will be seen that the pressure-induced thermal rejuvenation results in homogeneous deformation^22^.

The free volume is the difference between an atom's Voronoi volume and its atomic core volume. Since the latter is constant, the change in atomic volume may be expressed mathematically as:

6 $$\Delta \overline{V} = \overline{V} - \overline{{V_{0} }}$$

where $\overline{V}$ and $\overline{{V }_{0}}$ are the average atomic volume of a sample for a given value of ${P}_{R}$ and for ${P}_{R}=0$, respectively.

**Figure 3:**
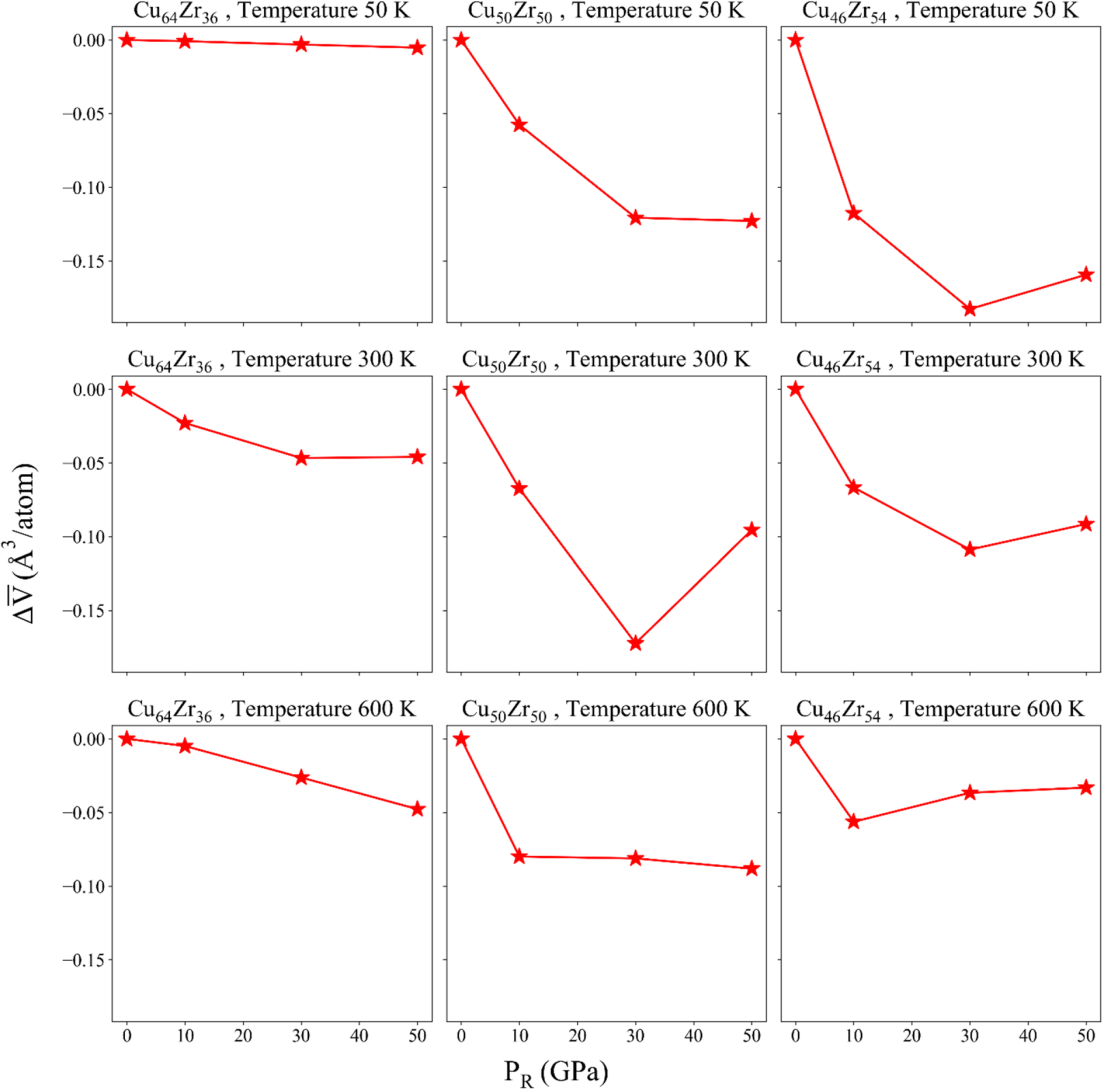
Variation of the atomic volume for various P_R_ values.

Figure 3 shows the change of atomic volume of each sample, for different ${P}_{R}$ values applied during the thermal-pressure treatments.

Based on the obtained results, it can be concluded that increasing the applied pressure up to 10–30 GPa (depending on the sample under study) during the thermal-pressure treatment, leads to a reduction in the volume of the samples. In other words, as the rejuvenation pressure increases, the structure becomes denser, indicating that pressure has a significant influence on changes in PE and atomic volume. Consequently, a high-energy state can be achieved alongside high density during the quenching process for each composition under different rejuvenation pressures. It is worth noting that higher annealing temperatures result in a greater degree of homogenization^44^, which is consistent with the findings presented in the next sections. This indicates a transition towards a more homogeneous structure. Furthermore, the increase in free volume is associated with a higher prevalence of loosely packed structures^44^. To further investigate this matter, it is crucial to examine the structural changes in the samples on a smaller scale, which will be discussed in subsequent sections. It is worth mentioning that in most of the cases under study, there is a minimum in the atomic volume at pressures around 10–30 GPa (depending on the atomic composition). The only exception is the Cu64Zr36 alloy. In a previous study, Amigo found that at large pressures (above 20–30 GPa), there is a loss of bond connectivity between high-centrosymmetric polyhedra, which leads to increased atomic volume^45^. The absence of the minimum in the Cu64Zr36 alloys can be explained from the increased number of Cu species. Given Cu is strongly related to high-centrosymmetric polyhedra, the prevalence of such species mitigates the reduction of bond connectivity.

#### Voronoi polyhedra

Amorphous solids, particularly MGs, are comprised of clusters, which are specific structural units. These clusters can be indexed geometrically via the Voronoi tessellation method^46,47^ and are essential for comprehending the structural behavior of MGs. The Voronoi tessellation is labeled by four indices $\langle {n}_{3},{n}_{4},{n}_{5},{n}_{6}\rangle$ where ${n}_{i}$ is the number of *i*-edged faces of the Voronoi polyhedron^48^.

As previously reported, Voronoi clusters (VCs) are divided into different groups, each of which is responsible for different properties in MGs^49^. Specifically, the $\langle 0,2,8,1\rangle$ and $\langle 0,3,6,3\rangle$ VCs are the relatively loosely-packed, while the $\langle 0,0,12,0\rangle$, $\langle 0,2,8,2\rangle$, and $\langle 0,1,10,2\rangle$ VCs are the full densely-packed clusters^50^. A high packing density indicates that the atoms in a material are tightly compressed, which decreases the free volume. This is due to the fact that the densely packed structure results in a high level of atomic coordination.

**Figure 4:**
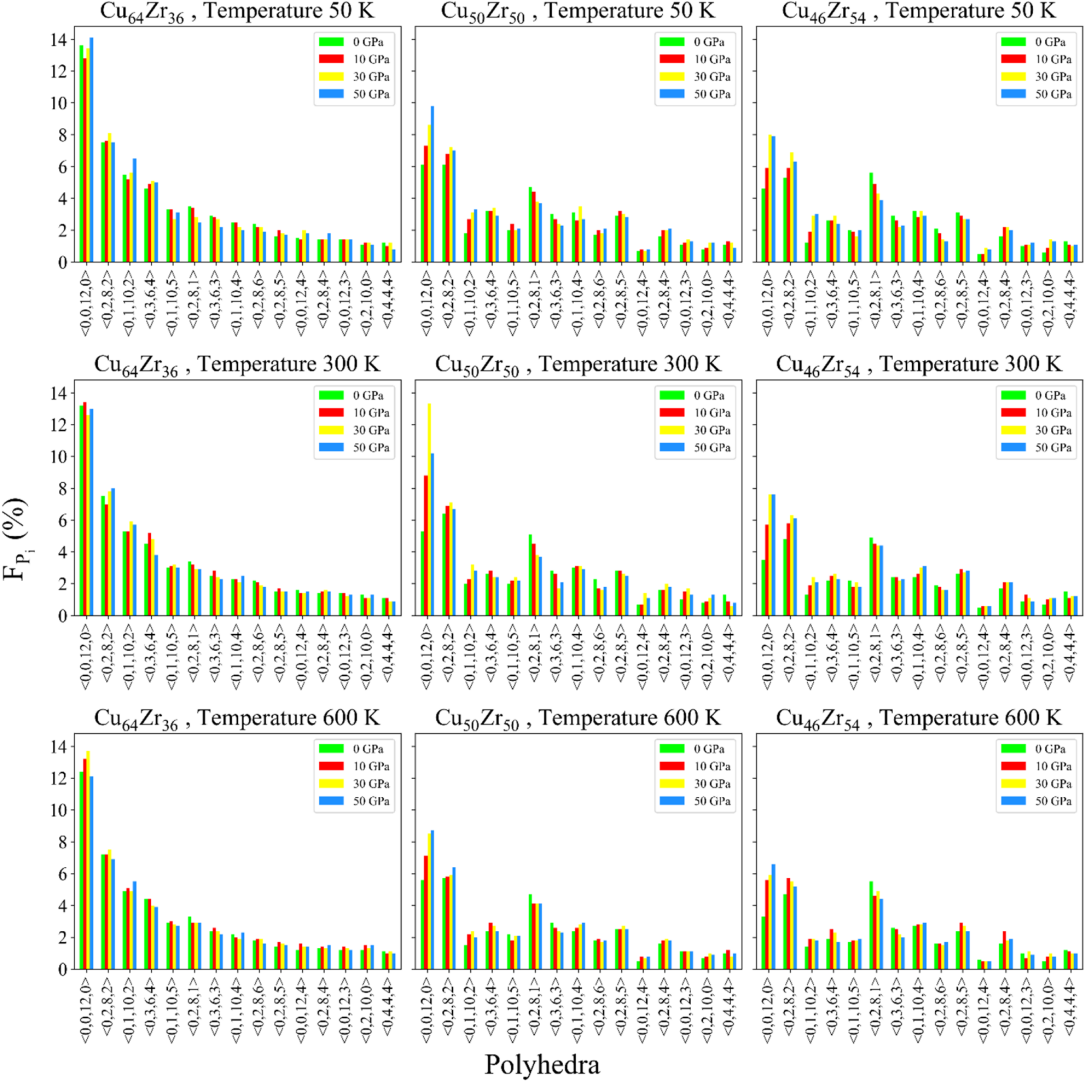
Voronoi population.

Here, as shown in Fig. 4, with increasing pressure, the fraction of $\langle 0,0,12,0\rangle$, $\langle 0,2,8,2\rangle$, and $\langle 0,1,10,2\rangle$ clusters increased. And on the other hand, the fraction of $\langle 0,2,8,1\rangle$ and $\langle 0,3,6,3\rangle$ clusters have decreased.

It can be said that with the increase of FIs, the free volume of the structure has decreased, which explains the decreasing trend of Delta V observed in Fig. 3. Actually, a more densely packed structure, as a result of pressure treatment, contains a greater proportion of FI, which is in complete agreement with the study of Wang et al.^25^. Previous work by Park et al. showed that, the larger amount of $\langle 0,0,12,0\rangle$ FI clusters is observed when the content of Cu is more^51^. As the amount of Cu increases, the fraction of $\langle 0,0,12,0\rangle$ polyhedra increases, which can be seen in Fig. 4. A higher Cu content leads to a larger yield stress, which is due to an increase in the population of the main FIs. In fact, FI clusters are resistant to structural deformations. A structure with more FI content has higher yield strength and fracture strength, which is related to more FI with higher Cu content^52^. This explains the larger yield strength displayed by the stress–strain curves of the Cu-rich samples. In the case of Delta V for the Zr-rich samples in Fig. 3, the decreasing trend is more noticeable, especially at 50 K. As observed from the VP analysis, these samples underwent a higher variation of densely-packed clusters, leading to lower free volumes. Nevertheless, the total population of such clusters is always lower than their Cu-rich counterparts.

### Mechanical response based on stress–strain curves

**Figure 5:**
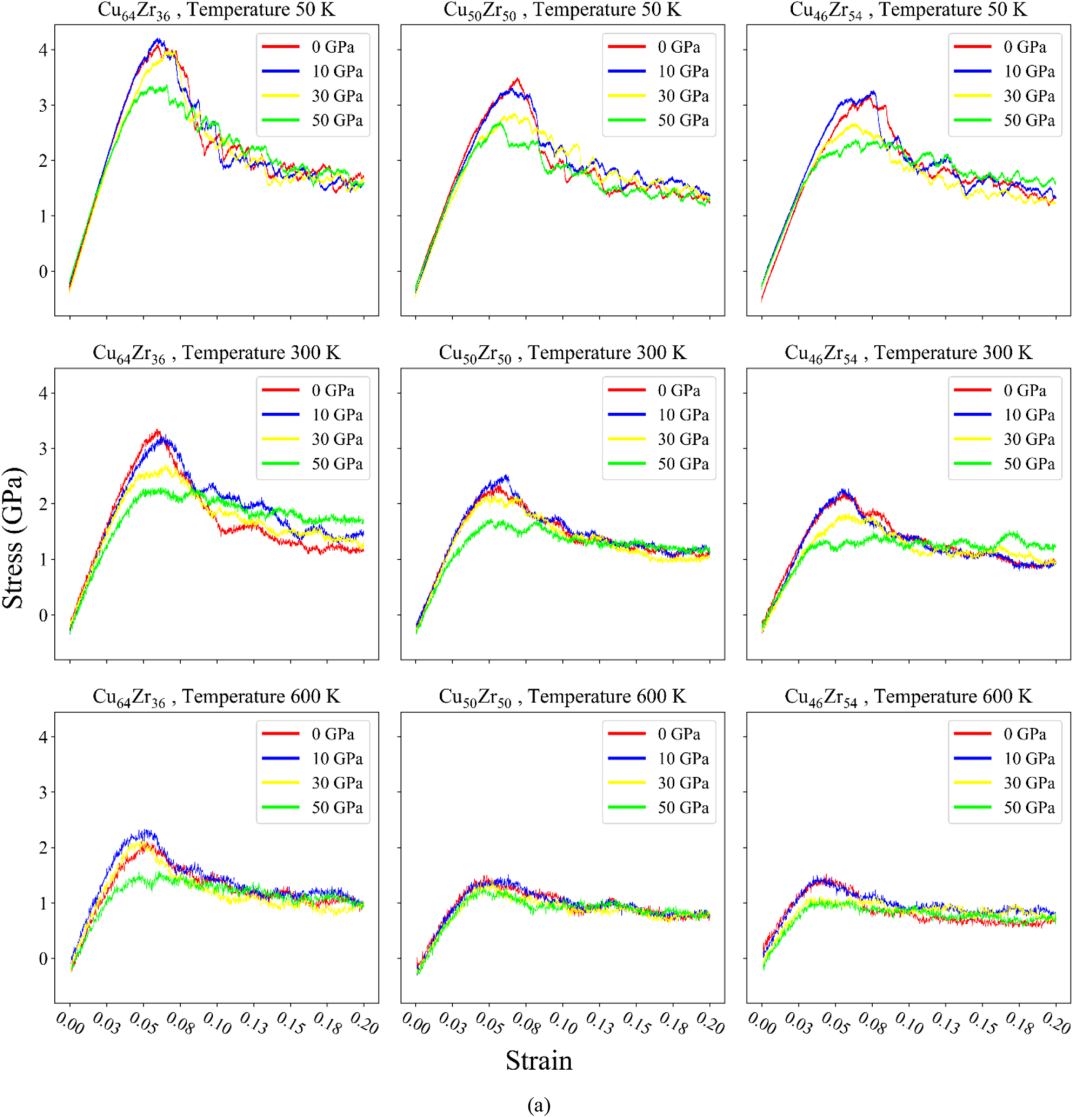

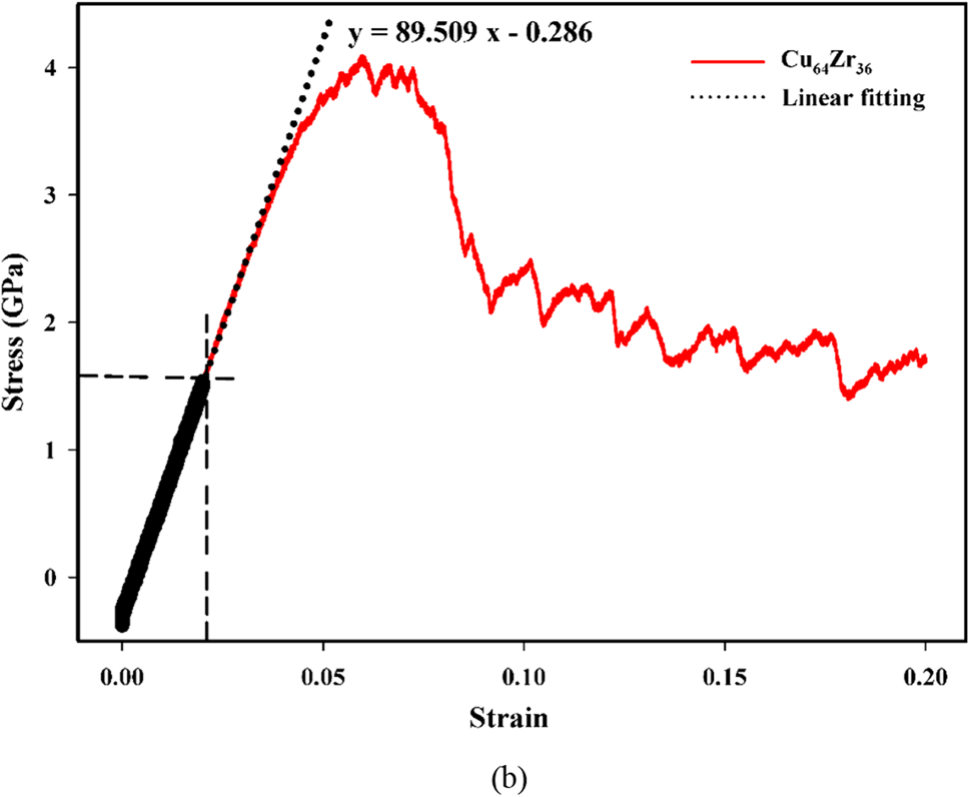
(**a**) Stress–strain curves for compression tests, (**b**) the stress–strain curve, fitting line, and equation for Cu_64_Zr_36_.

For MGs, it is crucial to understand the corresponding mechanical properties. The strain–stress curves resulting from compression tests are presented in Fig. 5a.

Figure 5a the data reveals that as the pressure is increased at a specific temperature and structure, there is a reduction in the yield stress. In all cases, a consistent pattern of elastic behavior is observed, with strains up to 0.02 as revealed from careful inspection of the curves. The inspection was carried out by fitting a straight line up to 0.02 strain. For better clarification, the stress–strain curve, fitting straight line, and equation for Cu64Zr36 are shown in Fig. 5b. This is followed by a phase of hardening, which continues until a strain of ~ 0.05–0.06 is reached. Beyond this point, a softening phenomenon occurs. Importantly, this behavior is found to be largely unaffected by the ${P}_{R}$. During the softening stage, an interesting observation is made in the 0 GPa sample, where there is a strong drop in stress at a strain of ~ 0.06–0.07. This behavior is commonly observed in MGs and is indicative of the initiation of localized deformation, a finding consistent with previous reports^4,53^. However, as the ${P}_{R}$ increases, particularly at pressures exceeding 30 GPa, this effect becomes less pronounced, suggesting a smoothing of the phenomenon. This finding implies a potential transition from localized to homogeneous deformation, aligning with previous findings^24^. This indicates that with increasing pressure under certain temperature and composition conditions, the system undergoes a transition towards homogeneity, resulting in enhanced ductility compared to its initial amorphous state which can refer to the rejuvenation of the MGs samples under pressure during preparation. As a result, through rejuvenation the system transforms from a brittle to a ductile state. Furthermore, for all structures at a specific temperature, it can be concluded that the final yield strength of the system increases with higher Cu content. Conversely, decreasing Cu content leads to the occurrence of the yield point at higher strains. Notably, as the amount of Cu decreases, less stress drop is observed and the structure moves towards more homogeneity. Consistently, as mentioned earlier, a noteworthy observation is that a lower Cu content corresponds to a more prominent softening phenomenon apparent in the stress–strain curves. Consequently, the degree of rejuvenation is also influenced by the Cu content, and different structures with varying Cu content exhibit different levels of rejuvenation^6^. It is worth mentioning that at a constant pressure for all compositions, lower temperatures yield higher final stress in the system, while increasing the temperature causes a decrease in the yield stress. Additionally, apart from pressure, it is evident that high temperatures also cause a lower stress drop, which consequently leads to a more homogeneous structure and results in the rejuvenation of the system^6^. As the temperature increases, the influence of compositions decreases, and this behavior is consistent across all systems. When comparing the effects of pressure and temperature on the stress drop, it can be concluded that temperature is more significant than pressure. This observation aligns with one of the distinctive characteristics of MGs, which exhibit exceptional strength at low temperatures and high flexibility at high temperatures^54^, validating our results.

Analyzing the changes in stress–strain curves along both rows and columns, it becomes evident that the behavior of different compositions becomes more similar at higher temperatures. Generally, as the temperature rises, the effect of compositions on the mechanical properties becomes more pronounced. With increasing temperature, the effects of other characteristics are attenuated at lower strains, and similar behavior is observed among different compositions. Lower temperatures have a lesser impact on the SRO structure of MGs. The SRO structure refers to the local arrangement of atoms within the disordered atomic structure of MGs. Unlike crystalline solids, which exhibit a highly ordered and periodic lattice arrangement of atoms, MGs possess a highly disordered and amorphous structure without long-range order. The SRO structures in MGs can have a detrimental effect on the mechanical properties at different temperatures^20^. It's worth noting that additional stress–strain curves were included in the supplementary material, which provide a better illustration of the impact of pressure, temperature, and material composition on the results.

### Microstructure evolution and degree of strain localization

**Figure 6:**
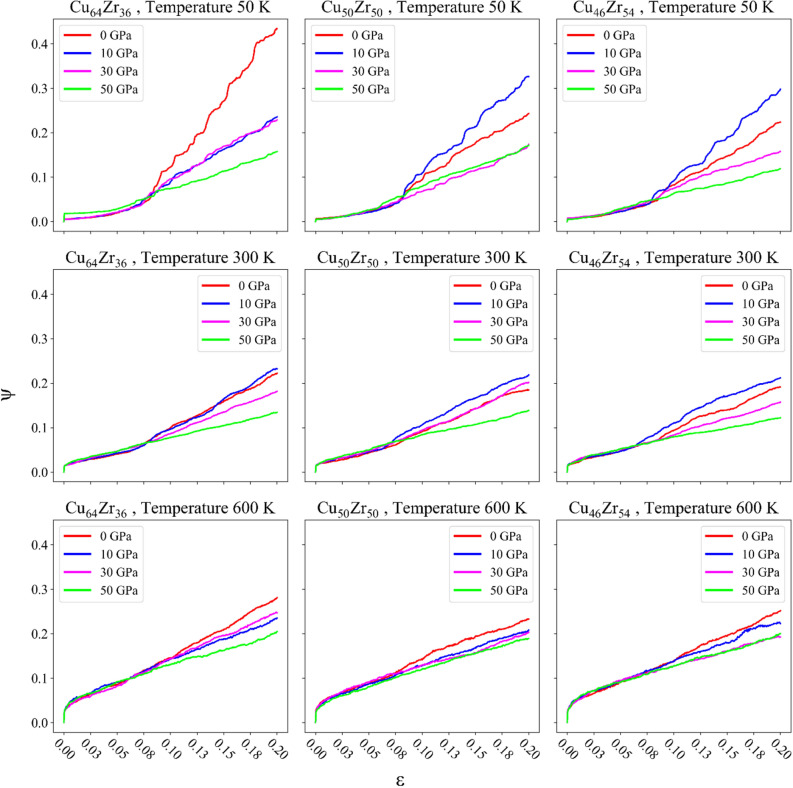
Degree of strain localization observed during the compression tests.

To examine the issue from another perspective, the degree of strain localization can be utilized. A larger degree of strain localization indicates a more localized mode of deformation^22^. The degree of strain localization is plotted as a function of strain for the rejuvenated samples in Fig. 6. In these curves, as the degree of strain localization increases, the structure moves towards localized deformation, while a decrease in strain localization signifies a shift towards homogeneous deformation. Thus, as the curves tend towards a straight line, a more homogeneous structure is achieved. Notably, the degree of strain localization demonstrates that the rejuvenated samples underwent localized deformation from 0 to around 10 GPa, and start to decrease for higher pressure, as indicated by the curve corresponding to 30 GPa, which tends towards a straight line across all structures. Additionally, according to the findings of this study, a transition from localized deformation to homogeneous deformation initiates at approximately more than 10 GPa.

**Figure 7:**
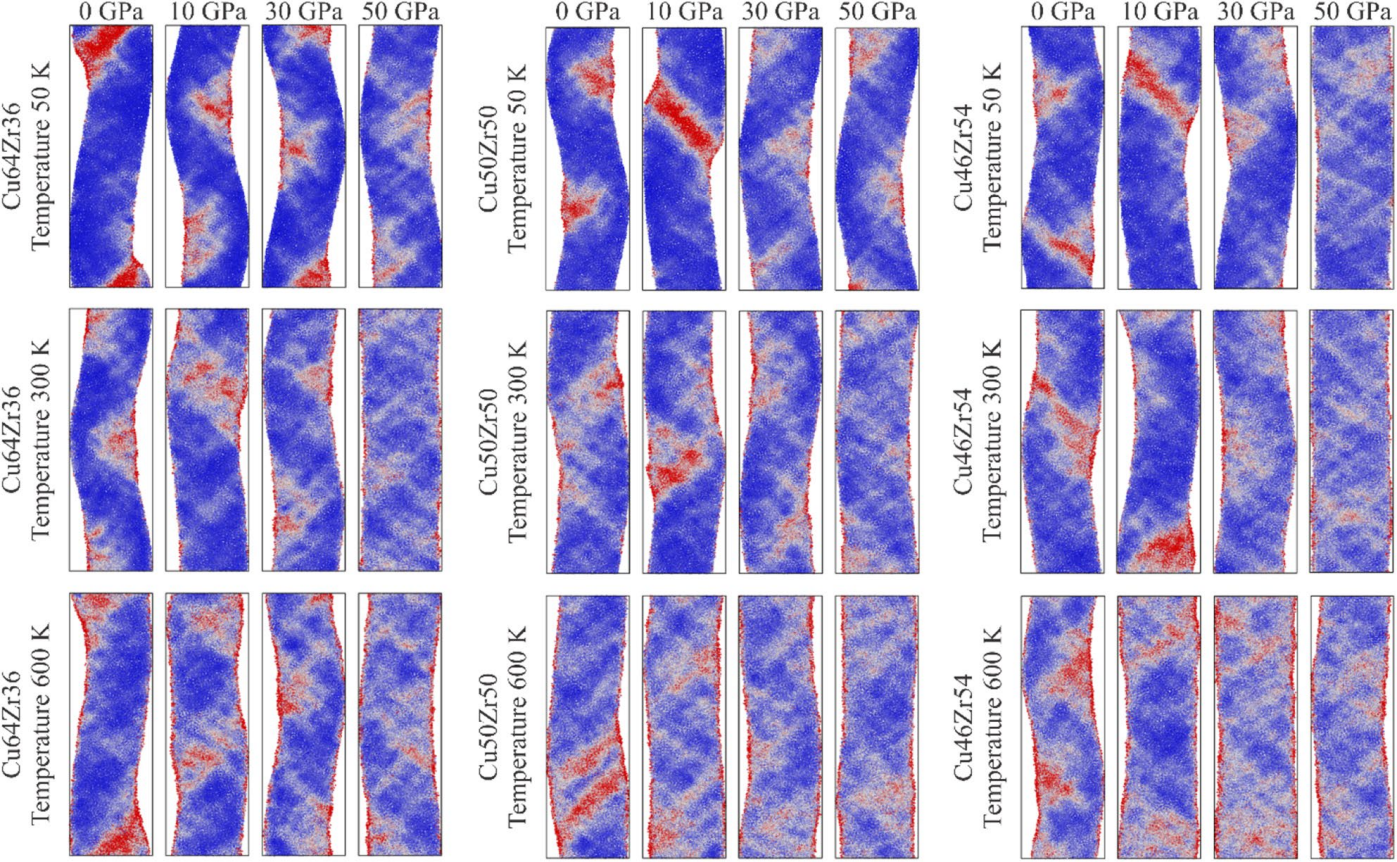
Transition from localized to homogeneous STZs nucleation for samples in the 0–50 GPa at $\varepsilon =0.12$.

To further investigate this transition, the local atomic von Mises strain is examined. By evaluating the atomic local shear strain, we aim to understand the underlying physical mechanisms responsible for the enhanced ductility observed in the samples. Figure 7 presents a series of snapshots illustrating the atomic deformation of rejuvenated samples at a strain of 12.0%. These snapshots allow for a detailed analysis of the atomic-level deformation processes taking place.

The snapshots reveal that a higher stress distribution corresponds to a more homogeneous structure. This is evidenced by the reduction in the concentration of red points and their dispersion, along with the increase in white points within the structure, indicating a progression towards homogenization. At 10 GPa, plasticity is primarily driven by the nucleation of localized STZs, while at 30 GPa, the nucleation of homogeneous STZs becomes more prominent. This observed trend aligns perfectly with the earlier findings, signifying that homogeneous plastic deformation occurs at 30 GPa. Furthermore, irrespective of the composition or pressure, it can be concluded that the system tends towards homogeneous deformation at high temperatures, as illustrated in Fig. 5a

Figure 5a it is worth noting that the initiation of this transition is contingent upon the structure, the preparation of the amorphous structure, the compositions, and the rejuvenation process. It is possible for similar transitions to occur at lower or higher pressures, as previously reported by Amigo and Valencia^5^, Ju and Zhou^55^.

### Elastic constants

**Figure 8:**
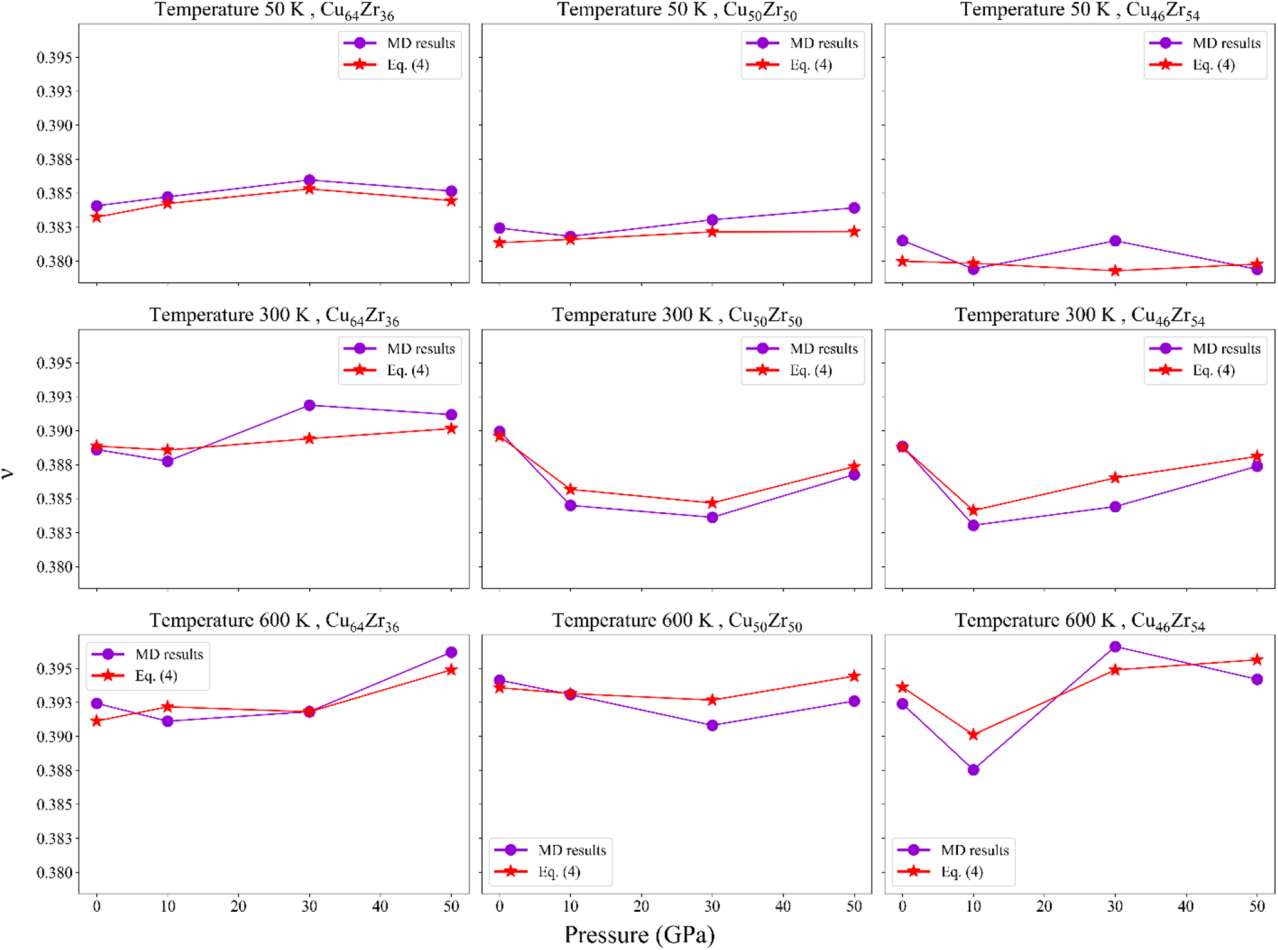
Poisson's ratio evaluated through MD simulations and by employing the mathematical relationship between $\nu$, B, and G.

The Poisson's ratio illustrates the relationship between the shape and volumetric deformation of a system. As used in earlier experimental investigations and MD simulations^24,56^, it was determined here to connect the atomic volume with the rejuvenation pressure. Figure 8 shows that Poisson's ratio has an increasing trend as reported by Amigo and Valencia^5^.

However, this process varies across different structures and temperatures. Generally, a high Poisson's ratio is indicative of good plasticity in MGs^56^. Increasing pressure in MGs leads to an increase in their Poisson's coefficient, and the distinct responses at each pressure reflect the structural and chemical bonding characteristics^56,57^. Additionally, the Poisson's ratio of MGs uniformly decreases with decreasing temperature^56^, as demonstrated by Fig. 8, and a higher value of Poisson's ratio promotes improved plasticity. The curve obtained using Eq. (4) closely follows the trend observed in the results obtained through MD simulations. At 10 GPa, as mentioned earlier, the structure remains in a state of localized deformation, which is also evident in Fig. 8. However, by increasing the pressure to 30 GPa, the structure undergoes a transition to homogeneous deformation, leading to an expected increase in Poisson's ratio. As Fig. 8 illustrates, Poisson's ratio increased for most of the structures at 30 GPa. This observation aligns completely with the findings presented in Figs. 6 and 7.

**Figure 9:**
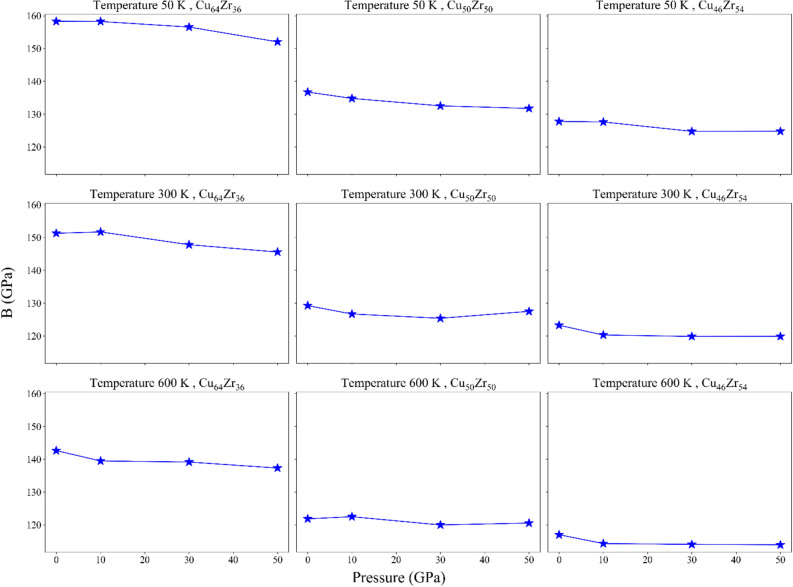
Bulk modulus for the rejuvenated samples.

**Figure 10:**
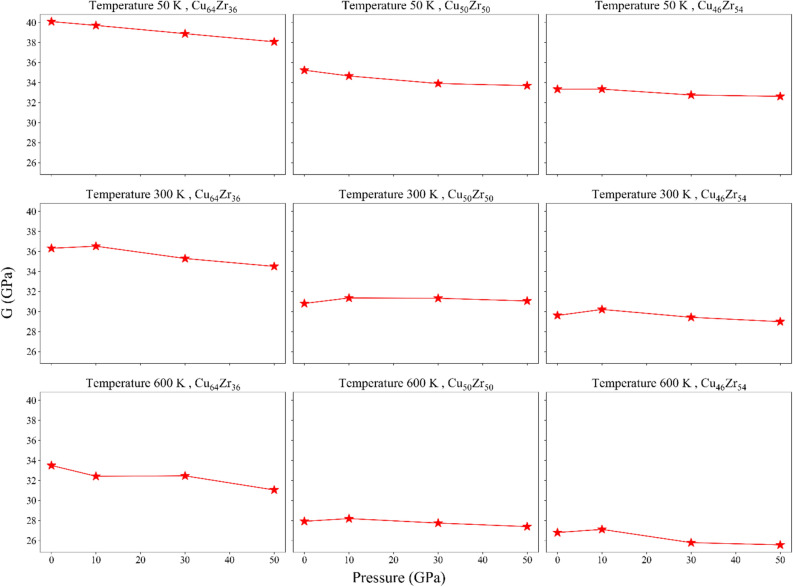
Shear modulus for the rejuvenated samples.

Figure 9 shows the bulk modulus and Fig. 10 shows the shear modulus, both of which have decreased.

This change in shear modulus and bulk can express the transition from shear banding to homogeneous plastic deformation. In fact, this is a confirmation of the relationship between elastic modulus and ductility in MGs^28^. The substantial drop in *G* is really compatible with the reduction in pure shear over hydrostatic contribution during yielding, which could perhaps lessen the STZs localization as shown in Fig. 6. In this approach, ${P}_{R}$ has a direct effect on the softening of *G* and, thus, on the contribution of deviatoric stress during the nucleation of STZs. However, it has little impact on *B*; temperature is the factor that matters most. So, it can be seen that the temperature has a direct effect on *B* and *G*, and as the temperature decreases, their values also decrease. Also, Cu content has a direct effect on the values of *G* and *B*, and has a similar behavior with the effect of temperature.

### Statistical description

In order to further understand the relationship between mechanical properties and those properties related to the synthesis of the sample, such as temperature (*T* = 50, 300, 600 K), Cu content (Cu = 46, 50, 64), and rejuvenation pressure (${P}_{R}=0, 10, 30, 50\,\mathrm{GPa}$), the Spearman correlation coefficient was calculated. This coefficient measures the degree of monotonic trend between two variables. A positive/negative value corresponds to an increasing/decreasing monotonic trend. The mechanical properties Young's modulus (*E*), Poisson’s ratio ($\nu$), shear modulus (*G*), and bulk modulus (*B*) were considered as variables. Six additional properties were also calculated following the work of Amigo et al.^58^: the yield stress (${\sigma }_{\gamma }$), resilience (${U}_{R}$), ultimate tensile stress (${\sigma }_{UTS}$), drop stress (${\sigma }_{D}$), flow stress (${\sigma }_{F}$), and toughness (${U}_{T}$). A brief description of each of them is given in the following: ${\sigma }_{\gamma }$ was obtained using the 0.002 strain offset criterion, ${U}_{R}$ is the area under the curve in the elastic regime given by the yield stress, ${\sigma }_{UTS}$ corresponds to the maximum stress, ${\sigma }_{F}$ is the average stress in the range of 0.15–0.20 strain, ${\sigma }_{D}$ is the difference given by ${\sigma }_{UTS}-{\sigma }_{F}$*,* and ${U}_{T}$ is the area under the whole stress–strain curve. The Spearman correlation coefficient for each pair of properties is defined as

7 $$\rho = 1 - \frac{{6\sum\nolimits_{i = 1}^{n} {d_{i}^{2} } }}{{n(n^{2} - 1)}}$$

where *d*_*i*_ is the difference between the two ranks of the pair of properties, and *n* is the number of mechanical tests (36 in our study).

**Figure 11:**
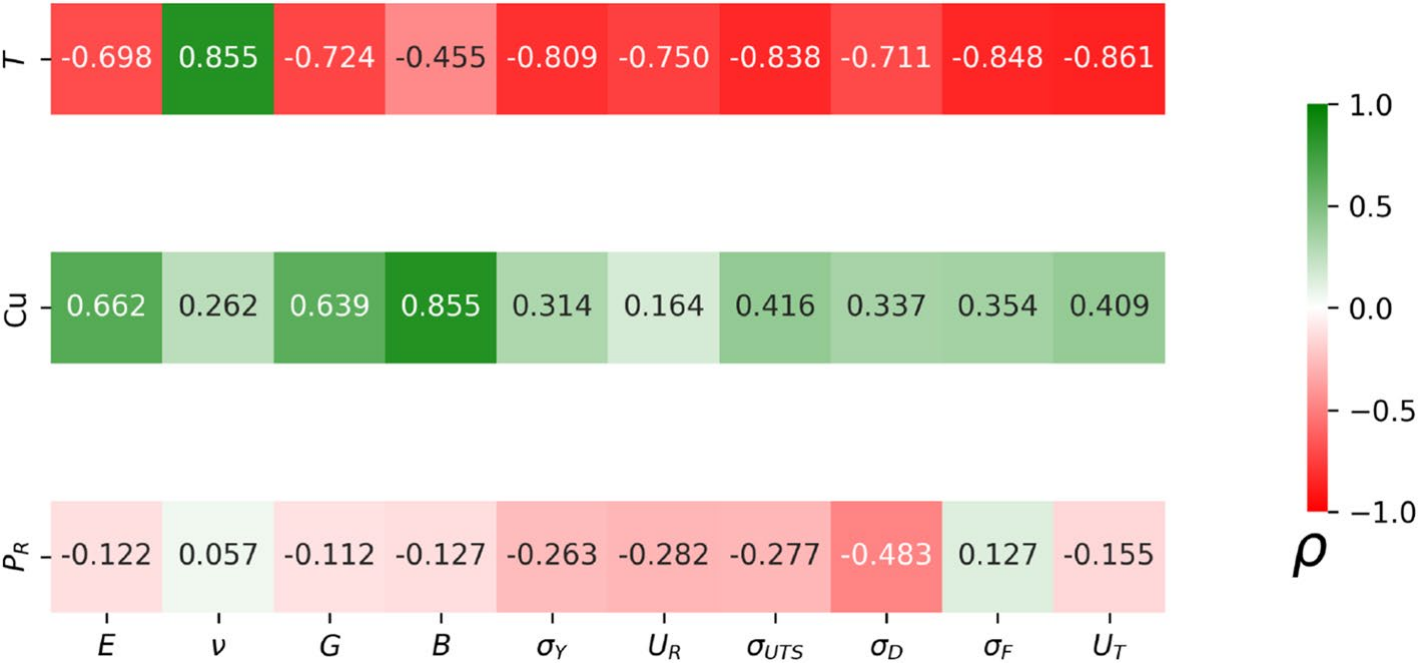
Spearman correlation coefficient (*ρ*) for temperature, Cu content, and pressure with each mechanical property.

The results of the Spearman correlation coefficient are shown in Fig. 11. In the case of temperature, a negative correlation is observed for all mechanical properties, as expected from the higher kinetic energy leading to enhanced atomic mobility and lower stiffness of the structural backbone of the MG. Poisson’s ratio is the only quantity that differs from this trend, since larger temperatures induce increased lateral strains which corresponds to lower stiffness. For Cu, all correlations show positive trends, with remarkable high values for *E*, *G* and *B*. It is well known that Cu atoms are associated with high-dense packed structures in MGs, strengthening the sample^59,60^. In the case of ${P}_{R}$, most of the correlations are almost negligible (values close to zero). An interesting exception is ${\sigma }_{D}$. Previous studies have shown that thermal-pressure treatments inhibit localized shear band formation, which is reflected from the decrease in drop stress^24^.

**Figure 12:**
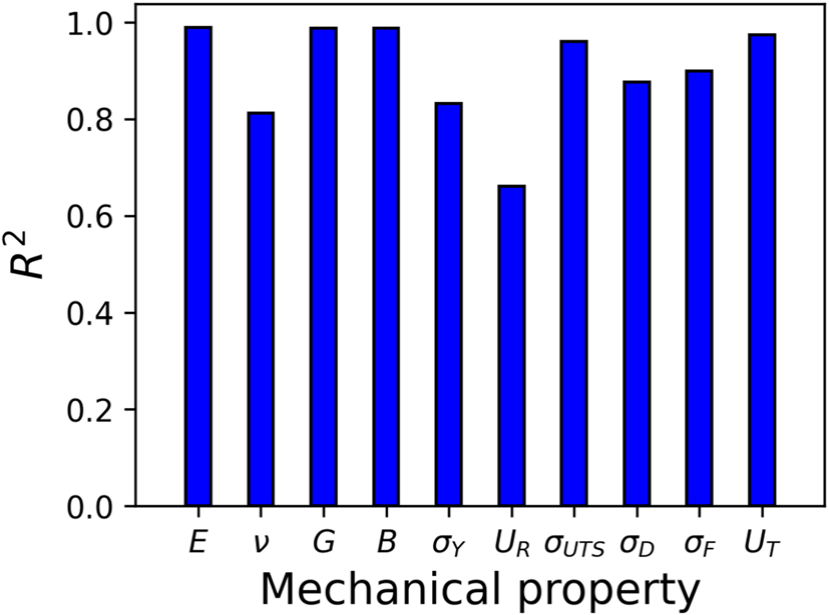
Coefficient of determination (${R}^{2}$) for temperature, Cu content, and pressure with each mechanical property.

Multivariate regressions were explored to establish models that relate mechanical properties with *T*, Cu and ${P}_{R}$. The regression models can be expressed as

7 $$y_{i} = B_{0,i} + B_{1,i} T + B_{2,i} Cu + B_{3,i} P_{R}$$

where ${y}_{i}$ stands for one of the 10 mechanical properties. The performance of each model was assessed by means of the coefficient of determination ${R}^{2}$, resulting in the values shown in Fig. 12. Overall, the models exhibit a remarkable performance above 0.6, indicating that the mechanical properties are strongly affected by temperature, atomic composition, and rejuvenation pressure. The lowest value corresponds to ${U}_{R}$, which can be explained from the methodology employed to calculate this quantity. The resilience was obtained as the area under the curve during the elastic regime, which in turn was determined using the yield stress and the 0.002 strain offset criterion. Therefore, ${U}_{R}$ depends on a second mechanical property, leading to higher statistical variability. Nevertheless, the performance of the models is promising, suggesting that the mechanical behavior of MGs can be predicted from the conditions used during their synthesis.

## Discussion and conclusion

We explored the mechanical properties of thermal-pressure rejuvenated Cu_x_Zr_100−x_ MGs. We conducted MD simulations to investigate the effects of temperature, pressure, and elemental composition on the mechanical response of MGs. The stress–strain response, elastic coefficients and moduli, degree of strain localization, PE, atomic volume variation, Voronoi analysis and microstructural evolution were analyzed to understand the effect of pressure and temperature on the rejuvenation process and mechanical properties of Cu_x_Zr_100−x_ MGs. The results of the MD simulations reveal important insights into the mechanical behavior of MGs. The strain–stress curves obtained from compression tests demonstrate a consistent pattern of elastic behavior, followed by a phase of hardening and then softening. Increasing the pressure leads to a reduction in the yield stress and a transition from localized to homogeneous deformation. This suggests that under certain temperature and composition conditions, the system undergoes rejuvenation, transforming from a brittle to a ductile state. The Cu content in the MGs also influences the mechanical response, with higher Cu content resulting in higher yield strength and stress drop. Temperature has a significant impact on the mechanical properties, with higher temperatures leading to decreased yield stress and more homogeneous structures. We discussed the importance of the rejuvenation process in MGs. Rejuvenation involves transitioning the system to a higher PE state, which can be achieved through various factors such as pressure, temperature, and composition. The results demonstrate that rejuvenation can be achieved by applying pressure during the preparation of MGs. The degree of rejuvenation is influenced by the Cu content, with different compositions exhibiting varying levels of rejuvenation. The findings also highlight the role of temperature in the rejuvenation process, with high temperatures leading to enhanced ductility and homogeneity in the system. We also utilized Voronoi analysis to validate our findings, which revealed increasing the pressure resulted in higher energy states and more dense atomic packing, primarily due to the significant increase in the number of $\langle 0,0,12,0\rangle$ polyhedra. Finally, a total of 10 materials properties were calculated and explored using statistical analysis to finding the correlation between temperature and atomic composition with mechanical properties. The results of the statistical analysis revealed distinct correlations between pressure, temperature, atomic composition, and mechanical properties, which suggest that the mechanical behavior can be predicted from such properties.

### Supplementary Information

Supplementary Information.

## Data Availability

The datasets generated during and/or analyzed during the current study are available from the corresponding author on reasonable request.
