# Supplementary material for: A molecular dynamics study on the mechanical response of thermal-pressure rejuvenated CuxZr100−x metallic glasses
Source: Sci Rep. 2023 Sep 26;13:16109. doi: 10.1038/s41598-023-43432-z (PMC10522610; doi:10.1038/s41598-023-43432-z)
Supplement: Supplementary file 1 — Supplementary Information. [file 41598_2023_43432_MOESM1_ESM.docx]

A molecular dynamics study on the mechanical response of thermal-pressure rejuvenated CuxZr100-x metallic glasses

**S. Sayad^1^, M. Khanzadeh^1^, Gh. Alahyarizadeh^1*^, N. Amigo^2^**

*^1^ Faculty of Engineering, Shahid Beheshti University, Tehran, Iran*

*^2^ Facultad de Ingeniería, Arquitectura y Diseño, Universidad San Sebastián, Bellavista 7, Santiago, 8420524, Chile*

** Corresponding author * E-mail:* [*g_alahyarizadeh@yahoo.com*](mailto:g_alahyarizadeh@yahoo.com)

Supplementary

Understanding the mechanical properties of metallic glasses (MGs) is essential. For this reason, the stress-strain curves that obtained from compression tests are presented in other views. Figure S. 1 exhibits temperature fluctuations within a stress-strain diagram, illustrating the impact of particular pressure and composition. Additionally, Figure S. 2 showcases the variations in composition at a specific pressure and temperature. The data illustrates that increasing pressure under a specific temperature and structure leads to a decrease in yield stress. Across all cases, a consistent elastic behavior is observed, with strains reaching up to 0.02. Subsequently, a hardening phase ensues, which persists until a strain of approximately ~0.05-0.06 is reached. Beyond this point, a softening phenomenon occurs. Remarkably, this behavior remains largely unaffected by the $P_{R}$. Notably, during the softening stage, an intriguing observation is made in the 0 GPa sample, where there is a significant stress drop at a strain of approximately ~0.06-0.07. This phenomenon, commonly observed in MGs, indicates the initiation of localized deformation, aligning with previous reports [1,2]. Nevertheless, with higher values of $P_{R}$, specifically when pressures surpass 30 GPa, the impact of this phenomenon diminishes, indicating a smoothing effect. This discovery suggests the possibility of a shift from localized deformation to homogeneous deformation, which is consistent with earlier research [3]. This indicates that, under specific temperature and composition conditions, increasing pressure prompts a transition towards homogeneity in the system. This transition leads to improved ductility compared to its original amorphous state, which can be attributed to the rejuvenation of the metallic glasses (MGs) samples during the pressure-induced preparation. As a result, the system undergoes a transformation from brittleness to ductility. Additionally, it can be deduced that, for all structures at a given temperature, the final yield strength of the system increases as the Cu content rises. Conversely, decreasing Cu content results in the occurrence of the yield point at higher strains. It is noteworthy that a decrease in Cu content is associated with reduced stress drop and a shift towards greater homogeneity in the structure. As mentioned earlier, this corresponds to a more pronounced softening phenomenon evident in the stress-strain curves. Hence, the degree of rejuvenation is influenced by the Cu content, and structures with different Cu content exhibit varying levels of rejuvenation [4]. It is important to note that, under constant pressure for all compositions, lower temperatures result in higher final stress within the system, whereas increasing temperatures lead to a decrease in yield stress. Furthermore, aside from pressure, it is evident that elevated temperatures also contribute to a reduced stress drop. This, in turn, promotes a more homogeneous structure and facilitates the rejuvenation of the system [4]. With rising temperature, the impact of compositions diminishes, and this behavior remains consistent across all systems. When assessing the effects of pressure and temperature on the stress drop, it can be inferred that temperature holds greater significance than pressure. This observation aligns with a notable trait of metallic glasses, which demonstrate remarkable strength at low temperatures and increased flexibility at high temperatures [5], thus corroborating our findings.

Upon examining the alterations in stress-strain curves along both rows and columns, it becomes apparent that the behavior of various compositions tends to converge at higher temperatures. Generally, as the temperature increases, the influence of compositions on the mechanical properties becomes more pronounced. At elevated temperatures, the effects of other characteristics are less prominent at lower strains, resulting in similar behavior across different compositions. In contrast, lower temperatures exert a lesser impact on the short-range order (SRO) structure of metallic glasses. The SRO structure pertains to the local arrangement of atoms within the disordered atomic structure of metallic glasses. Unlike crystalline solids, which possess a highly ordered and periodic lattice arrangement, metallic glasses exhibit a highly disordered and amorphous structure lacking long-range order. The SRO structures within metallic glasses can adversely affect the mechanical properties at varying temperatures [6].


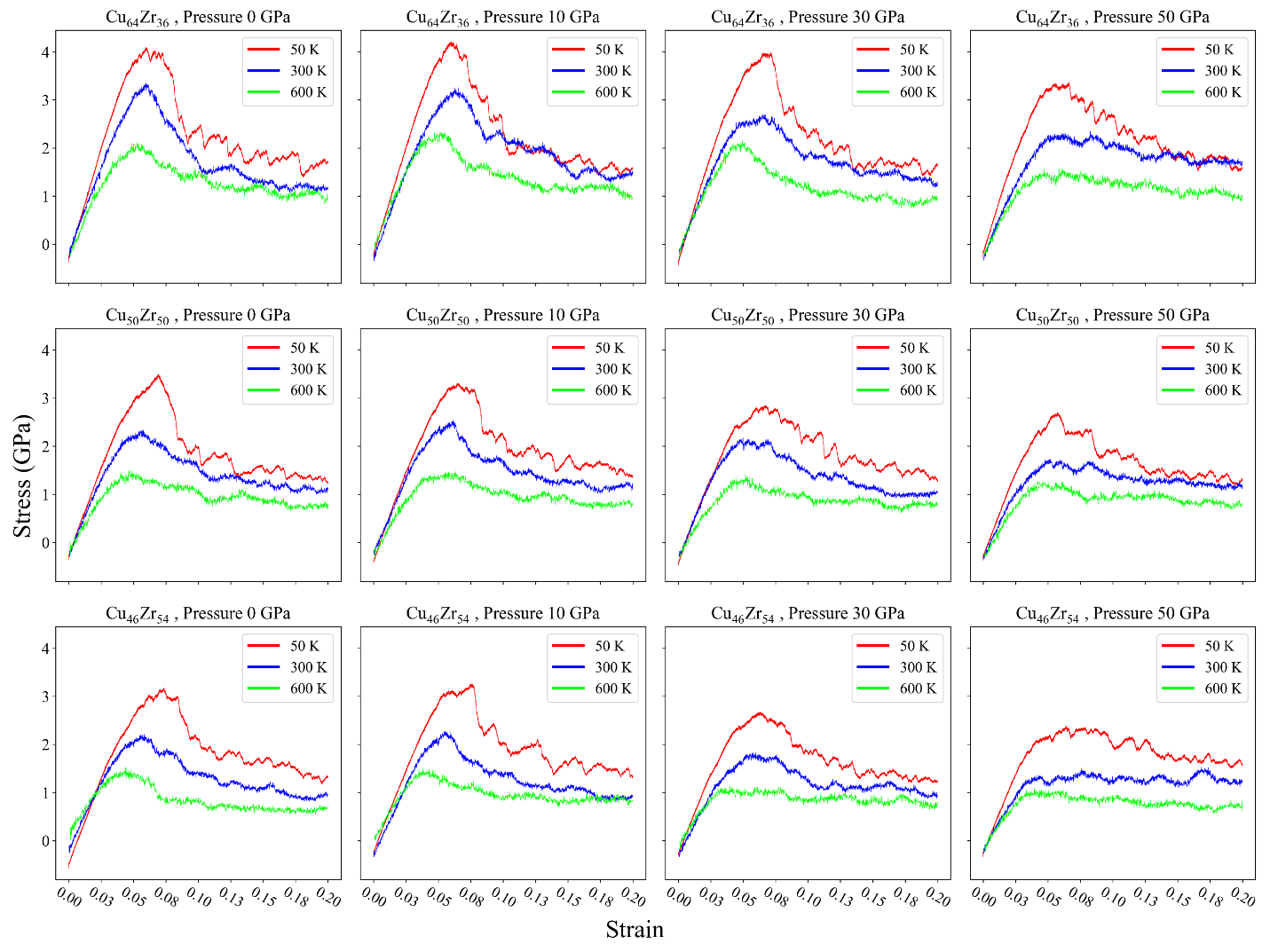


Figure S. 1- Stress-strain curves for compression tests


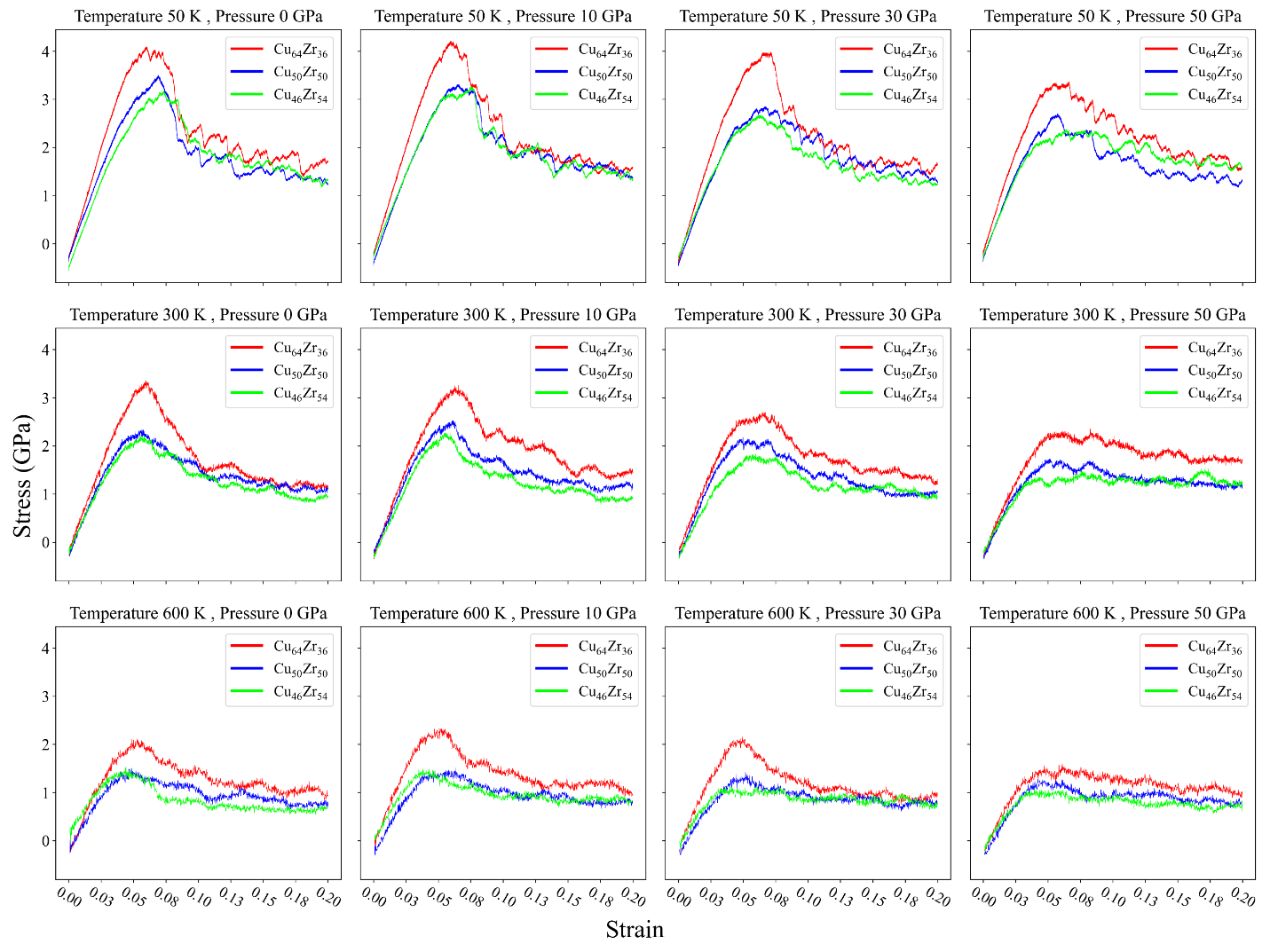


Figure S. 2- Stress-strain curves for compression tests

References

[1] Yue X, Brechtl J, Wang F, Chang Z, Liaw PK, Fan C. Deformation behavior of annealed Cu64Zr36 metallic glass via molecular dynamics simulations. Materials & Design 2020;191:108660. https://doi.org/10.1016/j.matdes.2020.108660.

[2] Zhong C, Zhang H, Cao QP, Wang XD, Zhang DX, Ramamurty U, et al. Size distribution of shear transformation zones and their evolution towards the formation of shear bands in metallic glasses. Journal of Non-Crystalline Solids 2016;445–446:61–8. https://doi.org/10.1016/j.jnoncrysol.2016.05.002.

[3] Feng SD, Chan KC, Zhao L, Pan SP, Qi L, Wang LM, et al. Rejuvenation by weakening the medium range order in Zr46Cu46Al8 metallic glass with pressure preloading: A molecular dynamics simulation study. Materials & Design 2018;158:248–55. https://doi.org/10.1016/j.matdes.2018.08.040.

[4] Wang P, Yang X. Atomistic investigation of aging and rejuvenation in CuZr metallic glass under cyclic loading. Computational Materials Science 2020;185:109965. https://doi.org/10.1016/j.commatsci.2020.109965.

[5] Chen M. A brief overview of bulk metallic glasses. NPG Asia Mater 2011;3:82–90. https://doi.org/10.1038/asiamat.2011.30.

[6] Hao H, Zhou W, Lu Y, Lau D. Atomic arrangement in CuZr-based metallic glass composites under tensile deformation. Phys Chem Chem Phys 2020;22:313–24. https://doi.org/10.1039/C9CP04914B.
